# Reduction in school individualized education program (IEP) services during the COVID-19 pandemic

**DOI:** 10.3389/fresc.2022.962893

**Published:** 2022-09-26

**Authors:** Belinda Chen, Patrick Rasmussen, Mallory Legg, Nicole Alexander, Pooja Vedmurthy, Akua Asiedu, Mihee Bay, Harolyn Belcher, Vera Joanna Burton, Charles Conlon, Amena Fine, Ryan Gill, Eboni I. Lance, Paul Lipkin, Joyce Wong, Anna Maria Wilms Floet, Sarah C. Doerrer, Jennifer Glattfelder, Amy Kordek, Julie Pertman, Rachel Murray, T. Andrew Zabel, Anne M. Comi, Mary L. Leppert

**Affiliations:** ^1^ Center for Development and Learning, Kennedy Krieger Institute, Baltimore, MD, United States; ^2^ Maryland Center for Developmental Disabilities, Kennedy Krieger Institute, Baltimore, MD, United States; ^3^ Department of Neurology, Kennedy Krieger Institute, Baltimore, MD, United States; ^4^ Department of Neurology and Developmental Medicine, Kennedy Krieger Institute, Baltimore, MD, United States; ^5^ Division of Neurodevelopmental Medicine, Department of Pediatrics, Johns Hopkins University School of Medicine, Baltimore, MD, United States; ^6^ Division of Child Neurology, Department of Neurology, Johns Hopkins University School of Medicine, Baltimore, MD, United States; ^7^ Department of Neuropsychology, Kennedy Krieger Institute, Baltimore, MD, United States; ^8^ Department of Psychiatry and Behavioral Sciences Johns Hopkins University School of Medicine, Baltimore, MD, United States

**Keywords:** COVID 19 pandemic, individualized education program (IEP), school services, remote/virtual education, compensatory services, recovery services

## Abstract

**Purpose:**

The COVID-19 pandemic created novel challenges for school systems and students, particularly students with disabilities. In the shift to remote/distance learning, this report explores the degree to which children with disabilities did not receive the special education and related services defined in their individualized education program (IEP).

**Methods:**

Patients attending an outpatient tertiary care center for neurodevelopmental disabilities in Maryland were surveyed on the impact of the pandemic on educational services provision.

**Results:**

Nearly half (46%) of respondents qualified for special education and related services through an IEP before the start of the COVID-19 pandemic. Among those with IEPs, 48% attested to reduced frequency and/or duration of special education and/or related services during the pandemic. The reduction was greatest in occupational therapy services (47%), followed physical therapy services (46%), and special education services (34%).

**Conclusion:**

This survey of children with disabilities observes a substantial reduction in IEP services reported in their completed surveys. To address the observed reduction in IEP services, we sought additional education for clinicians on the rights of students with disabilities in anticipation of students’ re-entry to the classroom. A special education law attorney provided an instructional session on compensatory education and recovery services to prepare clinicians to properly inform parents about their rights and advocate for patients with unmet IEP services during the pandemic.

## Introduction

IEPs were created as part of the Education for All Handicapped Children Act (EAHCA) in 1975, which mandated that local education agencies (LEAs) provide children with disabilities a free appropriate public education (FAPE) (1). IEPs require that expected levels of student academic and functional performance are monitored with measurable annual goals and define the least restrictive environment in which a student will receive special education and related services. According to the National Survey of Children’s Health, 8.9% of students in the United States (est. 6 million) received special education and related services (2). In October, 2020, 12.5% (110,569) of Maryland students were receiving special education services (3).

At the start of the COVID-19 pandemic, educational services were halted nationally for many students (4). The U.S. Department of Education (USDOE) issued guidance that if a local education agency (LEA) closes schools to help prevent the spread of COVID-19, and does not provide remote/distance learning for all students, there is no requirement to provide special education and related services for students with disabilities (5). Once school resumes in any form (e.g., in-person, remote/distance, or hybrid) the LEA must make every effort to provide special education and related services. The USDOE also clarified that schools can still meet legal obligations by providing equally effective alternative access during remote learning (6, 7). Despite this guidance, many LEAs have experienced difficulties in providing special education and related services during the COVID-19 pandemic (8). To meet these challenges, LEAs implemented plans to document the differences in the delivery of special education and related services between in-person and remote/distance instruction, provided remote/distance learning requirements, or provided checklists for educators amending IEPs during remote/distance learning (8). Recent literature has identified several student, parent, and teacher variables that impacted IEP implementation during the COVID-19 pandemic. The overarching purpose of this quality improvement project was to address the medical, social, emotional and behavioral needs of patients at clinic visits during the pandemic; however, this report is limited to the evaluation of the change in the provision of and access to special education and related services during the COVID-19 pandemic.

## Methods

Staff at an outpatient tertiary care center for neurodevelopmental disabilities in Maryland asked all patients to participate on a voluntary basis in a survey on the impact of the COVID-19 pandemic on medical and educational service provision and access. This data are gathered as part of a Quality Improvement Project and received IRB acknowledgement (IRB00262769).

Parents or guardians of patients under 22 years of age across all participating medical clinics completed questionnaires. For analysis, the data set was limited to respondents from one clinic, in which questionnaires included information about provision of educational services (*N* = 1,286). During the survey period, the 24 LEAs under the Maryland Department of Education independently determined whether education was provided in person, virtually, or as a hybrid. Further, some LEA’s may have begun the survey period with virtual instruction, but changed to in person or hybrid options, or switched venues as the community burden of COVID-19 increased.

To acquire COVID-19 related variables to inform outpatient clinical care, clinicians at Kennedy Krieger Institute (“the Institute”) initiated the use of a self-administered, pre-visit questionnaire to ask patients or patient parents or guardians about COVID-19 related concerns in advance of clinical visits. This survey was created on the Institute’s Qualtrics account and was distributed *via* web-link to patients across these programs prior to their in-person or telehealth clinic visits. The questionnaire collected information regarding the impact of COVID-19 on the patient’s health, behavior, medication, and treatment. The Institute’s Health Information Management department received the completed questionnaires through automated emails from Qualtrics and uploaded them to the health management system of EPIC. Clinicians were able to view the completed questionnaire before or during the clinical visit and addressed COVID-related patient concerns raised by the questionnaire during the clinical appointment. Race/ethnicity, sex, and diagnoses were extracted from EPIC for each patient for which a survey was received.

## Results

### Characteristics of survey respondents

A total of 1,286 parents or guardians were surveyed from June to December of 2020, with a response rate of 34%. Of the patients who responded, 70% were male; 51% were White, and 27% were Black or African American. Since access to educational services varied across states, patients living outside of Maryland were excluded, resulting in 1,168 surveys for analysis. Of the patients in Maryland, 70% were male, 49% percent were White, 29% Black or African American, 8% Asian, 2% Hispanic, 4% mixed/multiracial, and 5% identified as other. The average age of children was 9 years, 11 months (range 7 months to 24 years). From this population, 81% had a behavioral diagnosis, 54% had a developmental diagnosis, and 32% had a mental health diagnosis. The most common diagnoses were attention deficit hyperactivity disorder (ADHD) combined type (53.6%), mixed receptive and expressive language disorder (MRELD) (23.3%), ADHD inattentive type (16.2%), and anxiety (15.6%).

### Special education service delivery for patients with IEPs

**Figure 1:**
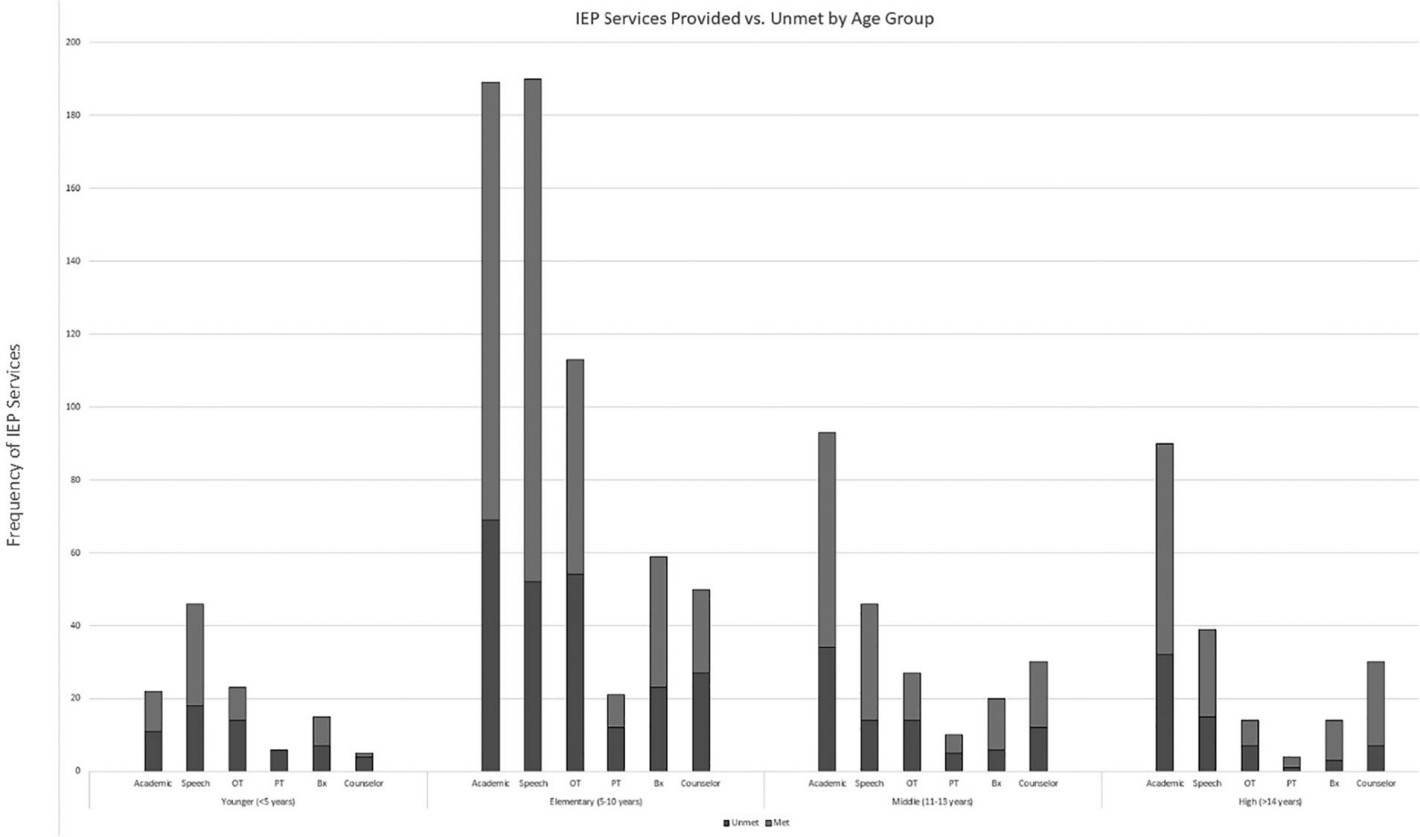
IEP services provided vs. unmet by age group.

Responses revealed that 538 (46%) patients had IEPs. The frequency of service provision for patients with IEPs was reduced for 259 (48%), maintained at the same level for 154 (29%), and increased for 35 (6%) patients who had IEPs. Unmet IEP services were noted to be greatest for occupational therapy services (47%) and physical therapy services (46%), while special education, speech-language, behavior, and counseling services were unmet for 30%–34% of patients (Table 1). There were no significant differences between the ratio of the frequency of IEP services provided and unmet across age groups (Figure 1).

**Table 1:** Attested frequency change of special education and related services during the COVID-19 pandemic (total students with IEP: *n* = 538).

Services indicated in IEP	Students receiving specific IEP service *n* (%)	Students who received specific service, and felt service need was unmet *n* (%)
Academic instruction (reading, math)	393 (73)	135 (34)
Speech and language services	322 (60)	98 (30)
Occupational therapy services	177 (33)	83 (47)
Physical therapy services	41 (8)	19 (46)
Behavioral therapy	108 (20)	32 (30)
School counselor/therapy	115 (21)	38 (33)
IEP but services not specified	20 (4)	0 (0)

### Barriers to attending remote/distance educational services

The survey included questions regarding the frequency of student attendance, barriers to attending remote/distance educational activities, and factors that influenced student participation in remote/distance learning in students with and without disabilities. Parents of children with and without IEPs stated that optional sessions, timing conflicts, and child refusal to participate were the most significant barriers that affected their child’s ability to participate in remote/distance learning sessions. These barriers may contribute to the explanation of decreased IEP services as well. Access to broadband and devices were not substantial barriers to participation for this cohort as only 0.4% endorsed these as barriers.

## Discussion

Prior to the pandemic, special education teacher shortages, teacher turnover, litigation, and staffing challenges in high poverty and rural schools led to shortages in special education services (9). The pandemic presented additional challenges to school systems, which particularly impacted children with IEPs. Nearly half of the children attending an outpatient tertiary care center for neurodevelopmental disabilities had a reduction in IEP service provision. The frequency of each type of unmet service was expectedly higher in services that traditionally would be more difficult to translate online, such as occupational therapy and physical therapy services.

LEA staff faced challenges of delivering services in the virtual setting for IEP services and accommodations that were provided during the pandemic. Some accommodations, such as preferential seating and physical prompting became irrelevant or impossible to implement during remote/distance learning. Other accommodations such as extended time and repeated directions prolonged time spent in remote/distance learning. These accommodations remained helpful for some students in the remote/distance learning setting, but did not yield the usual classroom-based benefit in the remote/distance learning environment for other students. In some situations, LEAs modified IEPs to employ a consultative model in which the provider coached the parent on the implementation of the targeted skill and the parent became the de facto teacher or therapist.

Balancing parental employment and household responsibilities with educational responsibilities, managing multiple school-aged children in the home, and navigating increased technical requirements may have led to parental stress and contributed to decreased access to special education and related services (10). Parental involvement is especially important during remote/distance education (11–13). A study from the Florida Southern College in 2012 indicated that the greatest parental concerns for remote/distance learning include keeping on schedule, self-discipline, and technical issues (12). During the COVID-19 pandemic, parents reported spending an average of 2.5 h a day on schooling (14). Remote/distance learning brought uncertainty to many parents as their role in their child’s education transitioned from overseeing homework activities to surrogate teacher or therapist (15). According to a survey from the University of Wisconsin-Stevens Point, lack of parent content knowledge, need for teacher communication, lack of access, and lack of online resource organization from teachers have caused accessibility issues during the COVID-19 pandemic (10).

As schools moved online, the challenges around COVID-19 may have led to lower student motivation and thus lower participation in educational services (16). Student motivation is an important factor in student success for remote/distance learning (17). A meta-analysis of kindergarten through grade 12 online learning literature found that the students who are usually successful in remote/distance learning are those “who had independent orientations towards learning, who were highly motivated by intrinsic sources, and who had strong time management, literacy, and technology skills” (18). Children with ADHD diagnoses, which made up two of the top diagnoses in this study, may find the more independent nature of remote/distance learning especially challenging.

Teachers also faced additional challenges during remote/distance learning. Prior research suggested that, from the teacher’s perspective, communication, lack of technology, and student participation are the biggest challenges for teaching online (19). Teacher interviews have also indicated that prior to the pandemic, remote/distance learning was a disappointing experience for some teachers compared to in-person learning due to lack of school and technical support, low student effort, and technology issues (20).

Well-designed IEPs are important in building skills that lead to success in the future of students with disabilities. Students who receive interventions to promote self-determination achieve education-related goals at a higher rate and have more positive community participation, employment, and quality of life after leaving school (21). Additionally, including students in the IEP meeting process promotes greater self-reliance and positive outcomes for the student (22).

Although parents, teachers, and other school staff have struggled to provide special education and related services during COVID-19, there was an opportunity to learn and improve from this experience. Increased funding to schools to support online learning helped mitigate access concerns. In March of 2020, the Federal Communications Commission (FCC) announced the Coronavirus Aid, Relief, and Economic Security (CARES) Act’s Education Stabilization Fund for remote learning. Through this grant, LEAs could purchase hardware, software, and connectivity access for remote/distance learning (23). Additionally, LEAs created professional development for teachers to address the differences in pedagogy between in-person and remote/distance learning. Due to the challenges and limitations during the COVID-19 pandemic, LEAs developed creative ways to build communities, train teachers remotely, and use remote tools such as asynchronous video and digital escape rooms (24). As students, parents, and educators work to improve access to special education and related services throughout the pandemic and beyond, it is important that the innovative spirit of problem solving continue to be fostered.

Given the stark contrast between the services documented in IEPs and services provided to students that were elucidated by this study, we sought instruction to improve clinicians’ ability to advocate for their patients. A special education law attorney provided education to the clinicians about compensatory education/recovery services in Maryland schools.

Compensatory education/recovery services attempt to place the student with an IEP in the position they would have been in had the student been provided with a free appropriate public education (FAPE). Each LEA will need to determine whether the educational services provided to the student during school closure and re-opening were reasonable to allow the student to make progress in the general education curriculum and on their IEP goals and objectives. Should the LEA find that compensatory education/recovery services are indicated, they must provide a plan to remediate the negative impact experienced by the student with IEP services due to the loss of FAPE (25).

Armed with accurate information regarding the process for negotiating or securing compensatory education/recovery services through LEAs, clinicians can advocate for their patients and counsel parents and guardians about strategies to access special education services and supports for their children.

## Limitations/future studies

These surveys were acquired over 7 months and analysis does not account for variations to access in the second year of the pandemic. Instead, the survey highlights the overall decrease in access to special education and related services from June to December of 2020. As part of this study, we did not inquire about the child’s primary disability or how the IEP correlated with our tertiary care center’s behavioral or mental health diagnosis. The study also included only English-speaking families, and did not account for variations in IEP services by specific LEA where instruction may have been delivered in person, virtually or in a hybrid model.

Further studies could look at the frequency with which clinicians addressed IEP concerns as students re-enter the classroom, and amount of compensatory education/recovery services awarded for students with IEPs post-pandemic. Additionally, it may be prudent to evaluate the students with IEPs that seemed to benefit from remote learning. Future studies could also investigate the relationship between the primary disability and the frequency and type of services provided for compensatory education/recovery services.

## Data Availability

The raw data supporting the conclusions of this article will be made available by the authors, without undue reservation.
